# Positive Selection on the Osteoarthritis-Risk and Decreased-Height Associated Variants at the *GDF5* Gene in East Asians

**DOI:** 10.1371/journal.pone.0042553

**Published:** 2012-08-14

**Authors:** Dong-Dong Wu, Gui-Mei Li, Wei Jin, Yan Li, Ya-Ping Zhang

**Affiliations:** 1 State Key Laboratory of Genetic Resources and Evolution, Kunming Institute of Zoology, Chinese Academy of Sciences, Kunming, China; 2 Laboratory for Conservation and Utilization of Bio-resource, Yunnan University, Kunming, China; Aarhus University, Denmark

## Abstract

GDF5 is a member of the bone morphogenetic protein (BMP) gene family, and plays an important role in the development of the skeletal system. Variants of the gene are associated with osteoarthritis and height in some human populations. Here, we resequenced the gene in individuals from four geographically separated human populations, and found that the evolution of the promoter region deviated from neutral expectations, with the sequence evolution driven by positive selection in the East Asian population, especially the haplotypes carrying the derived alleles of 5′ UTR SNPs rs143384 and rs143383. The derived alleles of rs143384 and rs143383, which are associated with a risk of osteoarthritis and decreased height, have high frequencies in non-Africans and show strong extended haplotype homozygosity and high population differentiation in East Asian. It is concluded that positive selection has driven the rapid evolution of the two osteoarthritis osteoarthritis-risk and decreased height associated variants of the human *GDF5* gene, and supports the suggestion that the reduction in body size during the terminal Pleistocene and Holocene period might have been an adaptive process influenced by genetic factors.

## Introduction

Humans are characterized by many unique traits, such as cognitive ability, language speaking, special skeletal anatomy, and susceptibility to diseases, which distinguish us from our closest relative, the chimpanzee (reviewed in [1]). In addition, modern humans exhibit substantial phenotypic variation, e.g., susceptibility to diseases, metabolism, skin pigmentation, eye and hair color, body mass, height, and craniofacial differences shaped by the skeletal system. Many studies have examined the genetic bases of the evolutionary patterns of these phenotypes and have identified the role of positive selection on genes in processes such as brain development in the human lineage and skin pigmentation among modern human populations (reviewed in [2], [3]). Similarly, in our previous studies we had concluded that positive selection in human skeletal genes had driven population differentiation in non-African populations [4], and identified a few skeletal genes that were subjected to this natural selection [5], [6]. To better understand the evolutionary forces acting upon skeletal genes, and associated traits, here we studied another critical skeletal gene, *GDF5*, in modern human populations.

GDF5 (growth differentiation factor 5) is a member of the bone morphogenetic protein (BMP) gene family and the TGF-beta superfamily and plays an essential role in the skeletal development. GDF5 is expressed in the primordial cartilage of appendicular skeleton, with little expression in the axial skeleton such as vertebrae and ribs [7], and is required for the normal formation of bones and joints in the limbs, skull, and axial skeleton [8]. Several kinds of skeletal disorders (e.g., acromesomelic dysplasia, Hunter-Thompson Type [9]; brachydactyly, type C [10]; chondrodysplasia, Grebe type [11]; fibular hypoplasia and complex brachydactyly [12]) are caused by mutations in the *GDF5* gene. The allele A of the SNP rs143383 in the 5′ promoter region of the *GDF5* gene was found to be associated with an increased risk of osteoarthritis, and shows decreased transcriptional activity of *GDF5* in chondrogenic cells [13]–[15]. In addition, this allele is associated with decreased height, which may be due to the lower expression of *GDF5* that could lead to a reduction in limb bone growth [16].

The functional importance of *GDF5* in skeletal development raises the possibility that this gene may contribute to the evolution of the human skeletal system. Evidence indicates that the human skeletal system has evolved rapidly since the advent of agriculture [17] suggesting that the selective pressures on skeletal genes changed during this process. Indeed, skeletal genes do demonstrate high population differentiation among different human populations, which was driven by positive selection [4]. The genetic basis, however, of the evolution of human skeletal system largely remains undocumented. Here we studied the population variation of the human *GDF5* gene by sequencing alleles from 142 individuals from four geographically separated populations from Africa, Europe, East Asia and South Asia. Positive selection was identified as operating on the 5′ UTR region of the gene in the East Asian population, with the target of selection being the derived alleles of the SNPs rs143384 and rs143383.

## Results

**Figure 1:**
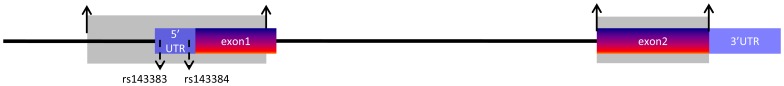
Structure of the human *GDF5* gene structure. The human *GDF5* gene is composed of two exons. The two regions sequenced in this study are denoted by the two rectangles.

Of the 284 chromosomes sequenced, 13 mutations were identified in the 1359 bp exon 1 region, which includes 5′ UTR and some coding sequences of *GDF5* (Fig. 1). To better study the sequence variation, we used the SNPs to construct haplotypes using the PHASE program [18], [19], and identified 16 haplotypes. Table 1 summarizes the population genetics data, including values for the nucleotide diversity, Tajima's D, Fu and Li's D, D*, F and F*, and Fay and Wu's H (see Materials and Methods). Population demographic history is the major confounding factor affecting the detection of positive selection. For example, negative values of Tajima's D can be attributed to population expansion, positive selection, or negative selection [2], [20], therefore, we used coalescence simulations, incorporating best-fit demographic parameters for the populations including European, African, and East Asian [21], to better understand the demographic histories of the populations. The results of the simulations indicated it was mostly positive selection rather than demographic effect that generated the variation of *GDF5*, although the factor of demographic effect can not be excluded absolutely. In the East Asian population, Fu and Li's D, and D* demonstrated significantly lower values with a *P*-value lower than 0.05. In the 875 bp sequenced exon 2 region 4 mutations were detected and 6 haplotypes were constructed. For this region the population variation did not deviate from the expectation of neutrality (Table 2). The observations for the exon 2 data support the hypothesis that positive selection, and not demographic history, operated on the 5′ UTR region, as demographic history should influence all parts of the gene similarly, and thus would be expected to produce the same pattern of polymorphisms in both the exons 1 and 2 regions. The difference in the patterns seen in the exon 1 and exon 2 regions thus means that demographic history cannot explain the exon 1 pattern.

**Table 1:** Population statistics summary of exon1 region.

Exon1 region (1359 bp, 12 SNPs)	Nucleotide Diversity	Tajima's D	Fu and Li's D	Fu and Li's F	Fu and Li's D*	Fu and Li's F*	Fay and Wu's H
African (N^a^ = 66)	6.209E-04	−0.451	0.052	−0.129	0.067	−0.115	0.703
European (N = 72)	7.954E-04	0.652	−0.207	0.077	−0.189	0.089	−0.176
East Asian (N = 76)	9.897E-04	−0.446	**−2.693**	−2.292	**−2.583**	−2.205	−0.217
South Asian (N = 70)	1.097E-03	0.470	0.230	0.365	0.239	0.370	0.865
Whole populations (N = 284)	1.052E-03	−0.604	−1.531	−1.422	−1.507	−1.404	0.761

The bolds are values significantly lower than 0 with P-value<0.05 by simulating human demographic history incorporating human best-fit model.

a is the number of chromosomes.

**Table 2:** Population statistics summary of exon 2 region.

Exon2 region (875 bp, 4 SNPs)	Nucleotide Diversity	Tajima's D	Fu and Li's D	Fu and Li's F	Fu and Li's D*	Fu and Li's F*	Fay and Wu's H
African (N = 66)	1.082E-03	0.976	−0.508	−0.059	−0.486	−0.042	0.386
European (N = 72)	5.665E-04	0.331	−1.011	−0.708	−0.991	−0.688	−0.333
East Asian (N = 76)	9.360E-04	0.639	−0.540	−0.204	−0.521	−0.188	−0.069
South Asian (N = 70)	9.488E-04	1.653	0.713	1.163	0.715	1.163	0.298
Whole populations (N = 284)	9.115E-04	0.409	−1.816	−1.273	−1.804	−1.263	0.132

**Figure 2:**
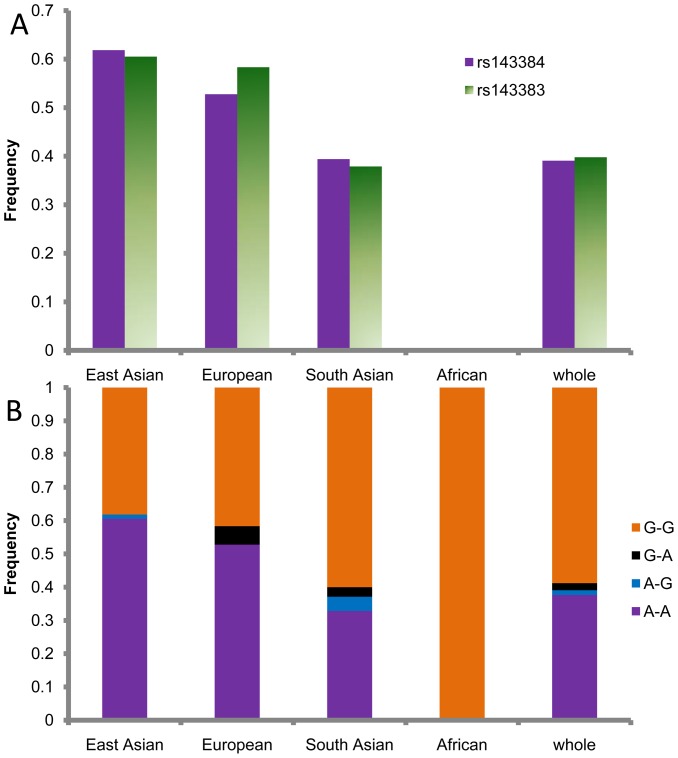
SNP allele frequencies in the *GDF5* gene. (A) Allele frequencies of haplotypes constructed using SNP rs143384 and rs143383 in four separate populations and the whole population. (B) Derived allele frequencies of the two SNPs in the four populations and the whole population.

**Figure 3:**
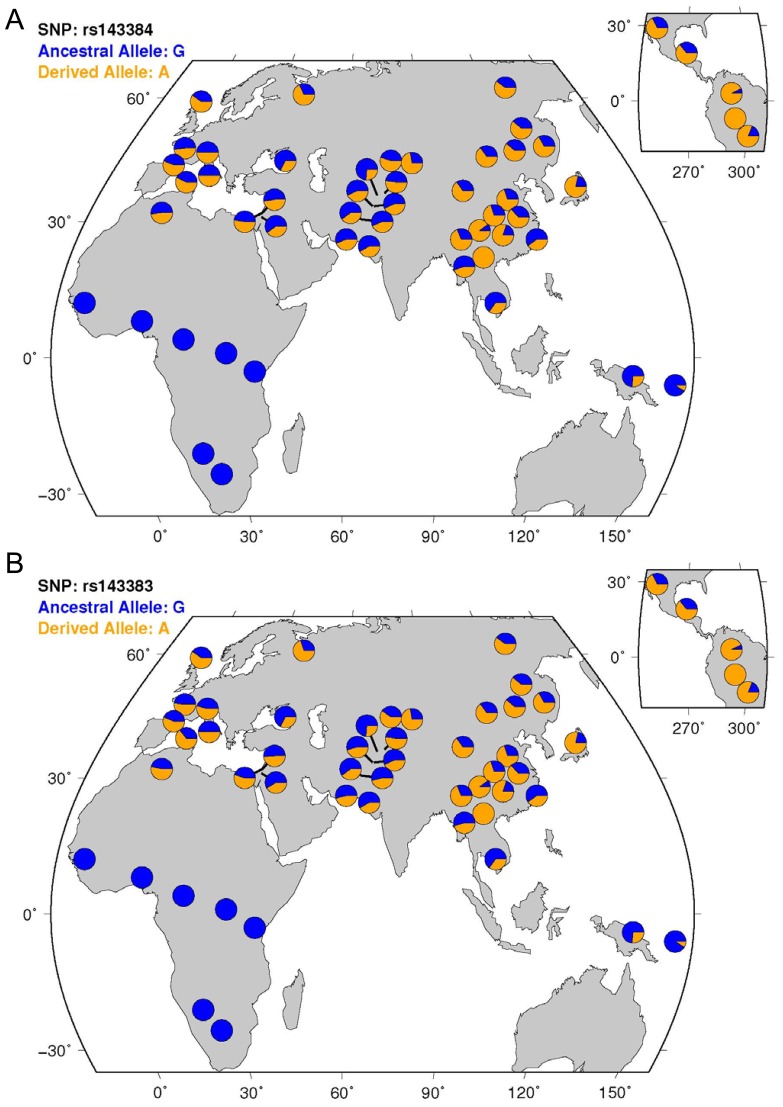
World-wide allele frequency distribution of the two SNPs rs143384 and rs143383. The data were downloaded from http://hgdp.uchicago.edu/.

Within the 5′ UTR region of the *GDF5* gene there are two SNPs, rs143384 (A/G, derived allele is A) and rs143383 (A/G, derived allele is A), which have high derived allele frequencies in the East Asian population (61.84%, and 60.53%, respectively, Fig. 2A). These two SNPs demonstrate significantly strong linkage disequilibrium (r^2^ = 0.857). We divided the 5′ UTR haplotypes into those carrying a derived allele and those carrying an ancestral allele of the SNPs. In the East Asian population only one haplotype carried the A-A pattern composed by the two derived SNP alleles and has a frequency of 60.53% (Fig. 2B). The high derived allele frequency was driven mostly by positive selection, to generate high haplotype homogeneity and was not destroyed by recombination. In contrast, the A-A haplotype is not found in our sequenced Africans (Fig. 2B). The World-wide allele frequency distribution also did not find the A-A allele in the sequenced Africans, despite the high derived allele frequencies in non-African populations (Fig. 3).

**Figure 4:**
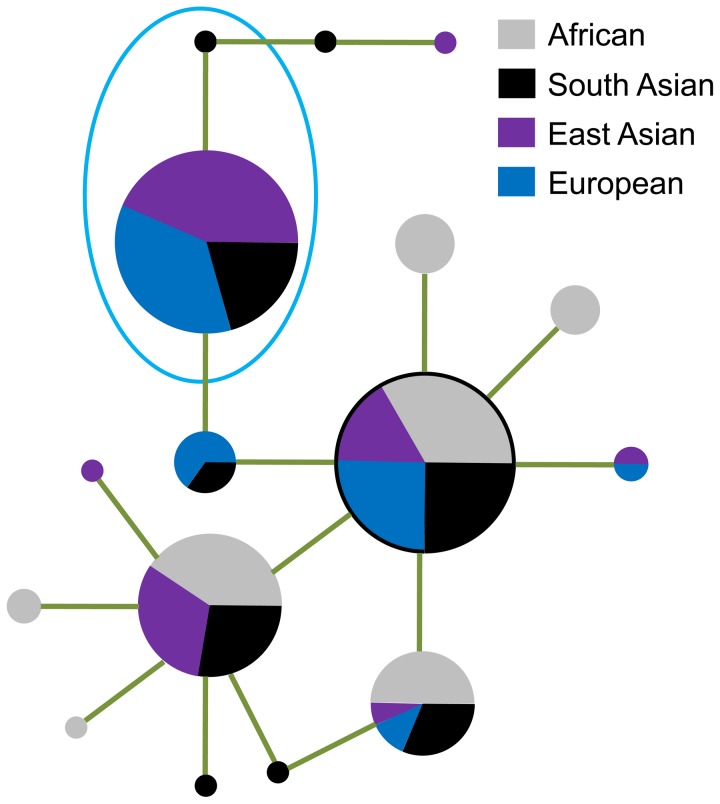
Median-joining phylogenetic network of the 16 haplotypes at at the exon 1 region. Each haplotype is represented by a circle with its area proportional to its frequency. The ancestral haplotype is outlined by a black line. The two haplotypes in the ellipse are the haplotypes that carry the derived allele at SNPs rs143383 and rs143384.

The derived allele A of SNP rs143384 is contained by two haplotypes, and these haplotypes demonstrate lower nucleotide diversity, lower Tajima's D, and significant lower Fu and Li's D* and F* (*P*<0.05) relative to haplotypes carrying the ancestral allele G (Table 3). The derived allele of the other SNP, rs143383, is contained by only one haplotype, which has a frequency of 60.53%. Haplotypes carrying the derived alleles also diverge from the others in the phylogenetic network (Fig. 4). In the Africans, there are no derived alleles at these two SNPs (Fig. 2, and Fig. 3), which indicates that these two SNPs were generated mostly after the “out of African” event. We also calculated the allele age of the derived alleles using the formula 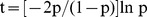, which considered that allele evolved under a model of neutral evolution [22], [23], where p is the allele frequency and t is age, measured in units of 2*N (effective population size) generations. With a generation time of 20–25 years and N = 10000, the ages of the derived alleles of SNPs rs143384 and rs143383 are 311,552∼389,440, 307,950∼384,937 years, respectively. These results suggest that the derived alleles could not reach the high observed frequencies (61.84%, 60.53%) under neutral evolution after the event of “out of Africa”, which only occurred about 100,000 years ago [24]. This suggests that positive selection may have been driving these two derived alleles to high frequencies.

**Table 3:** Population statistics summary of haplotypes carrying derived allele A and ancestral allele G of SNP rs143384.

rs143384 derived allele A	Allele Frequency	Nucleotide Diversity	Tajima's D	Fu and Li's D	Fu and Li's F	Fu and Li's D*	Fu and Li's F*
African (N = 0)	0	*N.A.*	*N.A.*	*N.A.*	*N.A.*	*N.A.*	*N.A.*
European (N = 38)^a^	52.78%	*N.A.*	*N.A.*	*N.A.*	*N.A.*	*N.A.*	*N.A.*
East Asian (N = 47)	61.84%	9.395E-05	−1.702	−1.752	−2.033	**−2.984**	**−3.026**
South Asian (N = 26)	39.39%	3.736E-04	−0.869	0.973	0.527	0.968	0.518
Whole populations (N = 111)	39.08%	1.302E-04	−1.493	−0.300	−0.811	−0.288	−0.798

rs143384 ancestral allele G	Allele Frequency	Nucleotide Diversity	Tajima's D	Fu and Li's D	Fu and Li's F	Fu and Li's D*	Fu and Li's F*
African (N = 66)	100%	6.209E-04	−0.451	0.052	−0.129	0.067	−0.115
European (N = 34)	47.22%	2.846E-04	−1.075	−0.341	−0.650	−0.302	−0.611
East Asian (N = 29)	38.16%	5.111E-04	−0.824	−2.100	−2.028	−1.958	−1.889
South Asian (N = 44)	60.61%	5.585E-04	−0.408	−0.090	−0.220	−0.064	−0.196
Whole populations (N = 173)	60.92%	5.421E-04	−1.230	−1.179	−1.431	−1.152	−1.407

Bolds are values significantly lower than 0 with P-value<0.05 detected by DnaSP v 5.0 program.

a European population contains only one haplotype carrying derived allele of rs143384.

**Figure 5:**
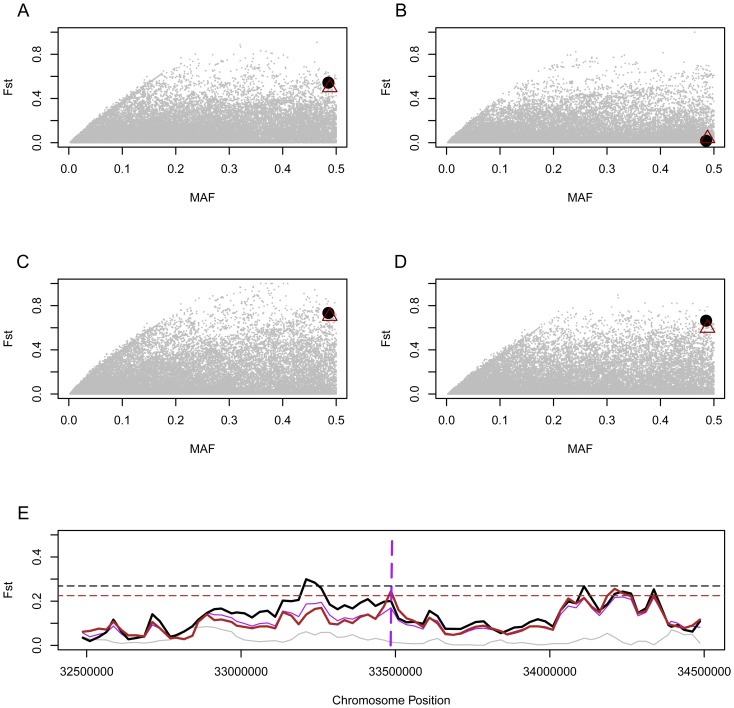
Fst distribution of SNPs across chromosome 20 gene regions. (A): Fst among the three populations vs minor allele frequencies (MAF). Big green dot and triangle represent SNPs rs143383 and rs143384. (B) Fst between East Asians and Europeans vs minor allele frequencies (MAF). (C) Fst between East Asians and Africans vs minor allele frequencies (MAF). (D) Fst between Africans and Europeans vs minor allele frequencies (MAF). (E) Sliding window analysis of Fst. Purple, black, brown and gray lines represent Fst among the three populations, Fst between East Asians and Africans, Fst between Africans and Europeans, Fst between East Asians and Europeans, respectively. The vertical line represents the position of *GDF5* gene. Black and brown horizontal lines represent the 95% percentile rank values of Fst values between East Asians and Africans and Fst between Africans and Europeans, respectively.

Further evidence for positive selection comes from the high population differentiation of the SNPs rs143384 and rs143383 among human populations. Here, we computed the Fst values of the SNPs based on three human populations using African (YRI), European (CEU), and East Asian (EA) data from HapMap to evaluate population differentiation. Fst values among the three populations at SNPs rs143383 and rs143384 are 0.544 and 0.499, respectively, which are higher than the Fst values of other SNPs in the gene regions of chromosome 20 (99.1%, 98.4% percentile rank) (Fig. 5A). Fst for the two SNPs between European and African are 0.664 and 0.597, for rs143383 and rs143384, respectively, values that are higher than the Fst values of SNPs in gene region of chromosome 20 (99.6%, 99.3% percentile rank) (Fig. 5D). Fst for the two SNPs between East Asian and African are 0.735 and 0.705, for rs143383 and rs143384, respectively, values that are higher than the Fst values of SNPs in the gene regions of chromosome 20 (99.5%, 99.3% percentile rank) (Fig. 5C). The Fst values of the two SNPs between East Asian and European, however, are not significantly higher (Fig. 5B). To further refine our analysis we performed a sliding window analysis of Fst values of other SNPs on chromosome 20 using a 50 kb window size and 25 kb step size. The Fst values of the two *GDF5* SNPs between Europeans and Africans are higher than the 95% percentile rank value for 50 kb regions of chromosome 20 (Fig. 5E).

**Figure 6:**
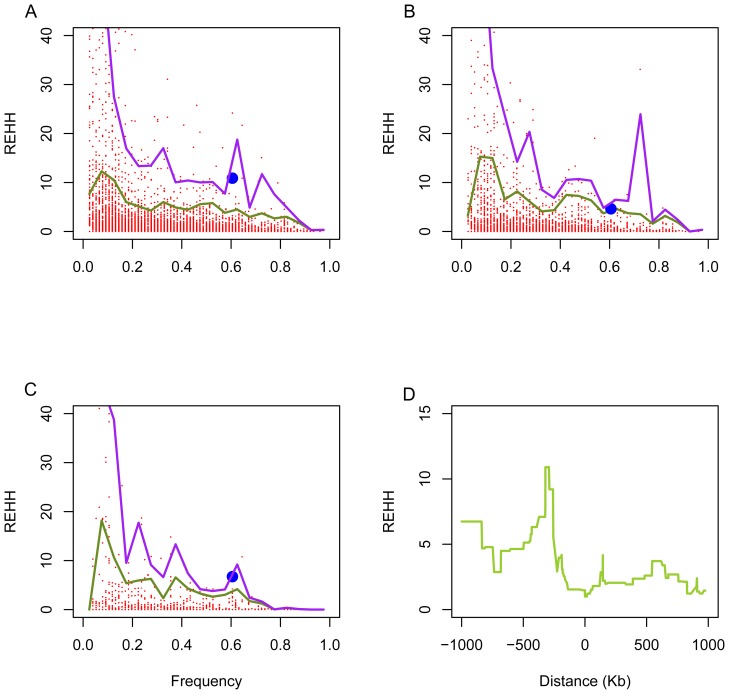
REHH of the core haplotypes on chromosome 20 and *GDF5*. REHH distributions at (A) 300 kb, (B) 500 kb, and (C) 1000 kb upstream and downstream of the core haplotypes. The two lines represent the 99% and 95% percentile rank values. Big dots are the major haplotype at the *GDF5* gene. (D) REHH of the major core haplotype at the *GDF5* gene at varying physical distances (kb).

To better understand the evolutionary pattern of *GDF5* in the human population we studied the extended haplotype homozygosity (EHH) of the *GDF5* exon 1 region in four populations, using the entire chromosome 20 phased haplotypes as empirical data. In the East Asian population, the major haplotype at the *GDF5* exon 1 and promoter core region (Fig. 4, haplotype in the ellipse), which contains the derived alleles of SNPs rs143383 and rs143384, reached 10.8986, 4.6377, and 6.7391 at 300 kb, 500 kb and 1000 kb upstream of *GDF5* core region, all of which are higher than the 95% percentile rank values (Fig. 6). These values support the conclusion that positive selection targeted the derived alleles of SNPs rs143383 and rs143384 in the East Asian population (Fig. 6).

**Figure 7:**
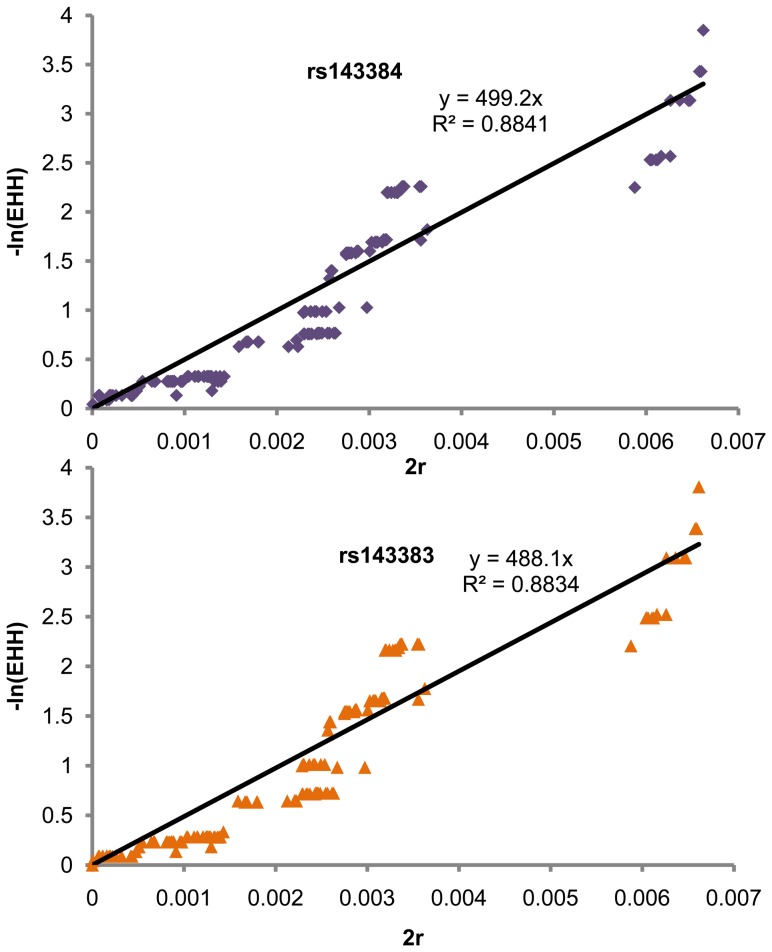
Evaluation of the ages of the derived alleles of SNPs rs143383 and rs143384 by linear regression of −ln(EHH) and 2r.

We employed an approach described in [25] to roughly estimate the ages of derived alleles of SNPs rs143384 and rs143383, using formula EHH≈Pr (Homozygosity) = e^−2rg^, namely, −ln(EHH)≈g*2r, where Pr(Homozygosity) is the probability that two chromosomes are homozygous at recombination distance r from the core, given identity by decent from a common ancestor g generations ago. Here, we used linear regression of −ln (EHH) and 2r to evaluate the value of g based on the EHH data in East Asian. As in Fig. 7, the age of derived allele of SNP rs143384, t = g*25 = 499.2*25 = 12,480 years, and the age of derived allele of SNP 143383, t = g*25 = 488.1*25 = 12,203 years.

## Discussion

**Figure 8:**
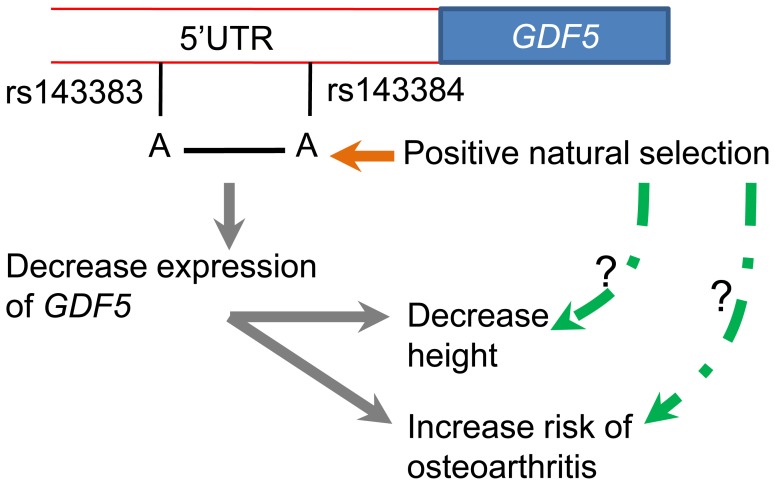
Positive natural selection operated upon the derived alleles of SNPs rs143384 and rs143383.

Our previous study indicated that positive selection operated on skeletal genes in non-African populations, including Europeans and East Asians [4]. Here, we describe positive selection acting in East Asian populations on a skeletal gene, *GDF5*, which plays a crucial role in the skeletal system. Positive selection probably targeted the derived alleles of SNPs rs143383 and rs143384 in the *GDF5* gene. The advantage of the derived alleles of these two SNPs is not clear. Strong evidence indicates that the derived allele of SNP rs143383 is associated with an increased risk of osteoarthritis, which is associated with decreased transcriptional activity of the *GDF5* gene in chondrogenic cells [13]–[16]. Lower expression of *GDF5* should lead to a reduction in limb bone growth and, as expected, the derived allele of rs143383 is associated with decreased height [16]. The two SNPs demonstrate significantly strong linkage disequilibrium, with the frequencies of the A-A and G-G haplotypes being 37.68% and 58.80%, respectively. The function of rs143383 on the expression of *GDF5* is influenced by the state of the rs143384 SNP [26]. Positive selection has driven the frequency of the derived alleles of these two SNPs to very high levels, leading to the associated decrease in height and increased risk of osteoarthritis (Fig. 8).

There is a decline in average human body mass, both in size and stature, began in the Late Pleistocene and early Holocene (∼12,000 years BP) [27], [28]. During this period, humans transited from lifestyle of close-contact ambush hunting of large mammals to the foraging and collecting of small animals. With the advent of agriculture, humans could produce food rather than needing to foraging for food [27], [28]. Technological improvements decreased the selective advantage of having a larger body, which is metabolically expensive to maintain. Nutritional inadequacies and the spread of infectious disease during the Holocene may also help explain the reductions in human body size [27], [28]. Changes associated with food production appeared to be developmental rather than genetic, however, the reduction in body size may also be due to genetic factors [27].

The ages of the derived alleles of SNPs rs143383 and rs143384 are ∼12,000 years supporting the hypothesis that the Late Pleistocene–Early Holocene decline in human body size results from a genetic factor that was driven by positive selection. Humans with smaller body size might have some advantages, and thus elevated probability of survival, due to the poor socio-economic conditions under nutritional stress [29], [30]. The decline in body size continued through the Neolithic, after which it was reversed in Europeans [27]. It had been concluded that when humans migrated to Europe they increased their body mass and height to facilitate their adaptation to this cold climatic area [31].

A question raised by our analysis is how can variants associated with diseases be positively selection for a fitness advantage? There are two main reasons to resolve this paradox. First, some characters that were adaptively evolved in the past may become maladaptive in a changing environment [3]. For *GDF5*, the derived alleles might have been positively selection for their advantage in the past, such as lower height, which would have increased survival in an environment with a lack of food. That advantage, however, may no longer be necessary. This would be similar to the example of the seven-repeat (7R) allele of the human dopamine receptor D4 (*DRD4*) gene. The 7R allele is associated with attention-deficit hyperactivity disorder (ADHD), however, people carrying this allele may have had an advantage in moving from one place to another during the colonization of world, and thus was driven to high frequency by positive selection [32], [33]. A second reason is gene pleiotropy. Pleiotropy means that a mutation that is advantageous in one instance can be unfavorable in another [3]. Osteoarthritis is probably a byproduct of the rapid evolution of human skeletal system. Furthermore, osteoarthritis is a disease associated with ageing, and is rare in individuals below the age of 45 years [14]. This means that the disease of osteoarthritis contributes very little to the fitness of the patient, as it only affects them after reproducing.

## Materials and Methods

### Sequencing of *GDF5* alleles in modern humans

*GDF5* gene sequences from a total of 142 unrelated human individuals, including 33 Africans, 36 Europeans, 38 East Asians and 35 South Asians, were chosen randomly from the Human Genome Diversity Cell Line Panel [34], were amplified by PCR and sequenced for two regions that include the two exons of the *GDF5* gene (Fig. 1). DNA sequencing was performed on an ABI 3730 automated DNA sequencer. Primer and PCR condition are available on request. Sequences were analyzed by DNAStar software. *GDF5* allele sequences of all individuals were submitted to GenBank under accession numbers GU831600–GU831883.

### Population variation analysis based on the re-sequenced data

SNPs detected in the resequenced *GDF5* alleles were used to construct haplotypes using the program PHASE [18], [19]. Median-joining network for the inference of haplotype genealogy was constructed by Network 4.5.1.0 [35]. The derived allele of each SNP was determined by comparing with the chimpanzee and orangutan sequences from UCSC genome database (http://genome.ucsc.edu/). Nucleotide diversity, which is the mean pairwise sequence difference, was calculated by the program DnaSP 5.0 [36]. A series population genetics parameters, Tajima's D [37], Fu and Li's D, F, D*, and F* [38], [39], and Fay and Wu's H [40], were used to measure deviation from neutrality in each population. Demographic history and natural selection can both generate similar patterns of population variation. For example, negative values of Tajima's D, Fu and Li's D, F, D*, F* can be due to either positive selection or population expansion. Accordingly, coalescent simulations were constructed that incorporate the best-fit demographic parameters, as described in [21], to calculate the significance of the deviation from neutrality.

### EHH analysis

Data on the genotypes of SNPs of chromosome 20 for the individuals that we resequenced for *GDF5* were downloaded from the Harvard HGDP-CEPH Genotypes for Population Genetics Analyses FLAT FILES SUPPLEMENT 10 from http://www.cephb.fr/en/hgdp/. We merged the SNPs data at *GDF5* to the genotyped data for chromosome 20 and constructed haplotypes for each chromosome using the fastPHASE program [41]. Positively selected alleles or haplotypes will quickly become accumulate, and tend to have strong extended haplotype homozygosity with surrounding loci as recombination would not have time to disrupt it [42]. Here, the extended haplotype homozygosity (EHH) and REHH (relative EHH) for each haplotype at 300 kb, 500 kb, and 1000 kb upstream and downstream of the core region were calculated by the Sweep program (http://www.broadinstitute.org/mpg/sweep/). In addition, the world-wide allele frequency distribution of the two GDF5 SNPs rs143384 and rs143383 was downloaded from the hgdp selection browser (http://hgdp.uchicago.edu/cgi-bin/gbrowse/HGDP/).

### Population differentiation analysis

Population differentiation of the SNPs on chromosome 22 was described in Wu and Zhang [43], which employed method from Weir BS and Cockerham [44], [45], and HapMap Phase II (release 24, NCBI36) [46] for the three populations: African (YRI panel including 60 Yoruban individuals from Ibadan), European (CEU panel including 60 individuals of Utah residents with ancestry from northern and western Europe) and East Asian (EA panels including 45 Han Chinese (HCB) and 45 Japanese from Tokyo (JPT)). A sliding window analysis was performed with a window size of 50 kb and a step size of 25 kb.
